# stTransfer enables transfer of single-cell annotations to spatial transcriptomics with single-cell resolution

**DOI:** 10.1016/j.crmeth.2025.101205

**Published:** 2025-10-15

**Authors:** Tao Zhou, Lin Xiang, Kuo Liao, Youzhe He, Zhenkun Zhuang, Shiping Liu

**Affiliations:** 1 College of Life Sciences, University of Chinese Academy of Sciences, Beijing 100049, China; 2 State Key Laboratory of Genome and Multi-omics Technologies, BGI Research, Hangzhou 310030, China; 3 School of Biology and Biological Engineering, South China University of Technology, Guangzhou 510006, China

**Keywords:** spatial transcriptomics, graph autoencoder, cell type transfer, Stereo-seq

## Abstract

Spatial transcriptomics (ST) enables *in situ* analysis of gene expression patterns and spatial microenvironments. However, current ST technologies are limited by detection sensitivity and gene coverage, posing significant challenges for precise cell type annotation at the single-cell level. To address this, we present stTransfer, a method that integrates reference single-cell RNA sequencing (scRNA-seq) data with ST context using a graph autoencoder and transfer learning. This approach minimizes information transfer loss between scRNA-seq and ST datasets. Benchmark analyses on publicly available spatial transcriptomic datasets demonstrate that stTransfer outperforms existing methods in both accuracy and robustness for cell type annotation. Lastly, we apply stTransfer to annotate neuronal populations in a high-precision Stereo-seq dataset of the zebra finch optic tectum.

## Highlights

• stTransfer enables single-cell annotation transfer to spatial transcriptomics
• Graph autoencoder plus VAE removes batch effects and preserves spatial context
• We demonstrate performance on four benchmark datasets across tissues and species
• We use stTransfer to annotate neurons linked to rapid visual memory in zebra finch

## Motivation

Current spatial transcriptomics (ST) technologies, while enabling *in situ* gene expression analysis, are constrained by limited detection sensitivity and incomplete gene coverage. These technical shortcomings severely impede accurate single-cell-level cell type annotation within spatial contexts. Existing computational methods attempting to transfer cell type information from single-cell RNA sequencing (RNA-seq) references to ST data inadequately address persistent batch effects, underutilize spatial location information, and lack the resolution required for precise single-cell annotation. These fundamental limitations necessitate the development of enhanced computational frameworks capable of integrating high-resolution single-cell annotations with spatial context while preserving biological accuracy.

## Graphical abstract

**Figure:**
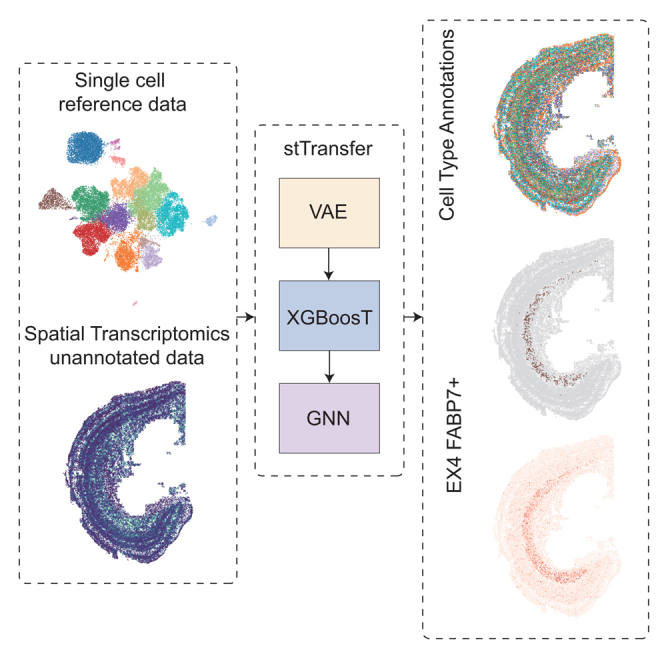

## Teaser

Zhou et al. present stTransfer, a transfer-learning framework that unites scRNA-seq and spatial transcriptomics to achieve single-cell-resolution annotation. Benchmarked on diverse datasets, it surpasses current tools and uncovers cell types in the optic tectum in zebra finch.

## Introduction

Single-cell RNA sequencing (scRNA-seq) has revolutionized our understanding of cellular heterogeneity, enabling detailed cell type annotation. Conversely, spatial transcriptomics (ST) provides insights into tissue-specific gene expression^1^^,^^2^^,^^3^^,^^4^^,^^5^^,^^6^^,^^7^^,^^8^^,^^9^^,^^10^^,^^11^ but struggles with resolution and gene coverage, limiting its ability to accurately annotate cell types.^12^^,^^13^ Bridging these gaps, we leverage transfer learning to combine high-resolution cell type information from scRNA-seq with spatial transcriptomics’ contextual data, achieving precise and spatially resolved annotations.

Although several methods have attempted to map cell type information from scRNA-seq to ST, such as Seurat,^14^ SingleR,^15^ and CellDart,^16^ which rely solely on RNA expression levels, and others such as Spatial-ID,^17^ RCTD,^18^ Cell2Location,^19^ Tangram,^20^ and DestVI,^21^ which incorporate spatial coordinates, these approaches face three key limitations: (1) inadequate handling of batch effects between scRNA-seq and ST data, (2) underutilization of spatial location information, and (3) insufficient resolution for annotating ST data at the single-cell level. To overcome these challenges, we propose stTransfer, a computational framework that integrates scRNA-seq and ST data using a variational autoencoder (VAE), effectively leveraging spatial embeddings while mitigating batch effects for high-resolution, spatially precise cell type annotation.

In this study, we propose stTransfer, a computational framework that integrates scRNA-seq and ST data to achieve high-resolution, spatially informed cell type annotation. stTransfer addresses three key challenges: (1) batch effects between scRNA-seq and ST data, (2) transfer of cell type information from scRNA-seq to spatial data, and (3) incorporation of spatial context for precise annotation. To achieve this, stTransfer employs a three-step strategy: first, it utilizes a VAE^22^ to harmonize batch effects between scRNA-seq and ST data. Second, it trains an XGBoost model^23^ on finely annotated scRNA-seq data to predict cell type distributions in spatial data. Finally, it performs graph embedding on ST data to capture spatial relationships between cells and their neighbors, enabling context-aware cell type annotation. By combining these steps, stTransfer not only improves the accuracy of cell type transfer but also provides spatially resolved annotations at single-cell resolution, offering a powerful tool for exploring tissue architecture and cellular interactions.

## Results

### The pipeline of stTransfer

**Figure 1:**
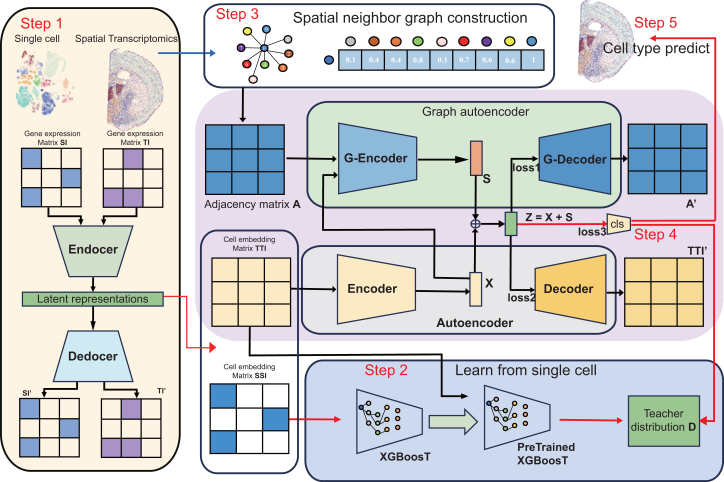
Overview of stTransfer Step 1: input the single-cell gene expression matrix SI and the ST gene expression matrix *TI*. Perform reconstruction training through a variational autoencoder (VAE) to obtain the cell embedding matrix SSI for single-cell data and the cell embedding matrix TTI for ST data. Step 2: use the cell embedding matrix *SSI* as a reference for training the XGBoost classifier model. Input the cell embedding matrix *SSI* into the pre-trained XGBoost classifier to obtain the teacher distribution *D* learned from the single-cell data. Step 3: input the spatial position information of each cell in the ST data. Construct the graph embedding by calculating the relationships between spatial cells to obtain the adjacency matrix *A*. Step 4: input the cell embedding matrix *TTI* into the encoder of the autoencoder to obtain *X*. Input *X* and the adjacency matrix *A* into the graph encoder of the GAE to get *S*. Combine *X* and *S* to get the final latent representations *Z*. Reconstruct the cell embedding matrix *TTI′* using the decoder of the autoencoder and reconstruct the adjacency matrix A′ using the graph decoder of the GAE. Additionally, train a classifier with the final latent representations Z and the teacher distribution *D* obtained in step 2. Step 5, re-input the cell embedding matrix *TTI* and the adjacency matrix *A* into the encoder of the autoencoder and the graph encoder of the GAE to obtain *Z*, and input *Z* into the classifier to predict the final cell type of each spatial single cell.

stTransfer employs a three-step framework to achieve high-resolution, spatially resolved cell type annotation (Figure 1). Each step leverages distinct aspects of the data to ensure precision and biological relevance.

#### Batch effect correction via VAEs

To mitigate batch effects between scRNA-seq and ST data, stTransfer employs a VAE. The VAE encodes gene expression matrices from both datasets into low-dimensional latent representations, capturing shared biological variability while minimizing technical discrepancies. Specifically, the encoder maps single-cell and spatial transcriptomic matrices into latent embeddings, which are then reconstructed by the decoder to optimize performance. This step ensures unified embeddings that preserve biological signals while harmonizing batch effects.

#### Cell type annotation transfer using XGBoost

With harmonized embeddings, stTransfer transfers cell type annotations from scRNA-seq to ST data. The latent embedding derived from single-cell data trains an XGBoost classifier, which learns the cell type distribution from annotated scRNA-seq data. This trained model predicts probabilistic cell type distributions for spatial cells, bridging the gap between the two modalities.

#### Spatial context integration using graph embedding

To incorporate spatial context, stTransfer constructs a graph embedding based on cell adjacency. An adjacency matrix is derived from Euclidean distances, where closer cells exhibit stronger connections. The latent embedding undergoes graph encoding, combining gene expression and spatial adjacency to generate spatially informed embeddings. These embeddings are optimized using reconstruction losses for gene expression and adjacency matrices, alongside classification losses based on teacher distributions. This ensures spatially coherent and accurate annotations.

stTransfer integrates batch effect correction, cell type transfer, and spatial context modeling to achieve high-resolution, spatially resolved cell type annotation. We tested its performance on four benchmark datasets: (1) simulated pseudo data, (2) STARmap-sequenced mouse brain data,^24^ (3) CosMx SMI-measured human lung cancer data,^25^ and (4) Slide-seq-sequenced mouse spermatogenesis data.^26^ To demonstrate its practical utility, we applied stTransfer to a Stereo-seq dataset of the zebra finch optic tectum (OT), revealing distinct neuronal populations and showcasing its ability to analyze complex tissues.

### stTransfer outperforms competing methods in annotating single-cell resolution pseudo-ST data with complex tissue patterns

To rigorously evaluate stTransfer’s performance on single-cell resolution ST data, we performed extensive benchmarking using two types of simulated pseudo-ST datasets. These datasets were meticulously designed to replicate real-world biological scenarios: one modeled hierarchical tissue organization (e.g., brain cortical layers) and the other represented block-like structures (e.g., tumor regions). Both datasets included four distinct cellular components along with background noise, effectively capturing the complexity and heterogeneity typical of real biological samples.

**Figure 2:**
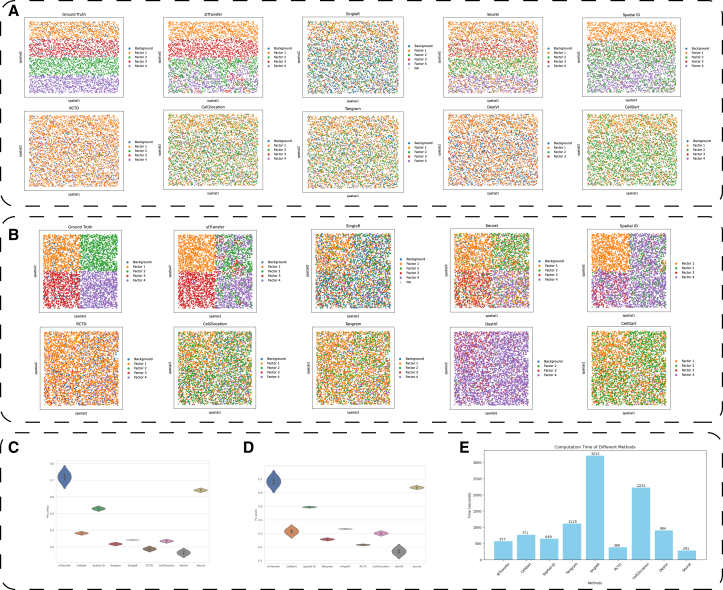
Performance comparison of stTransfer and other methods on pseudo ST data (A and B) Spatial plot showing the cell type predicted by different methods on the pseudo hierarchical and block structures, respectively. Each point represents a cell, and its color indicates the predicted cell type. The ground truth is shown in the top left corner of each panel. (C and D) Violin plots comparing the weighted-F1 score and accuracy of different methods on the pseudo hierarchical and block structures, respectively. (E) Bar chart showing the computation time required by different methods. The *x* axis lists the methods, and the *y* axis shows the computation time in seconds.

In the hierarchical structure dataset, stTransfer demonstrated outstanding performance (Figure 2A). It achieved an impressive annotation accuracy of 70%, surpassing all other methods evaluated (Figure 2C). Notably, DestVI exhibited the lowest performance, with an accuracy of only 27.3%. When classification precision was assessed using the weighted-F1 score, stTransfer retained its leading position with a score of 68%, while DestVI lagged significantly at 16.7% (Figure 2D).

The method’s superiority was further validated in the block structure dataset analysis (Figure 2B). Here, stTransfer achieved its peak performance metrics, with an accuracy of 74% and a weighted-F1 score of 70.6% (Figures 2C and 2D). Seurat ranked second with an accuracy of 63.4% but still trailed behind stTransfer. Besides, stTransfer without the VAE module can achieve a mean accuracy of 62.7% on the 2 samples; this shows that the VAE module may play a very important role in stTransfer. Importantly, despite its advanced functionality, stTransfer maintained computational efficiency, with processing times only slightly longer than those of RCTD and Seurat (Figure 2E).

These findings highlight stTransfer’s robust ability to accurately annotate complex ST patterns at single-cell resolution while maintaining computational efficiency. Its consistent outperformance across various tissue organization patterns underscores its potential as a powerful tool for ST analysis in diverse biological contexts.

### stTransfer achieves superior accuracy in annotating high-resolution mouse brain ST data

**Figure 3:**
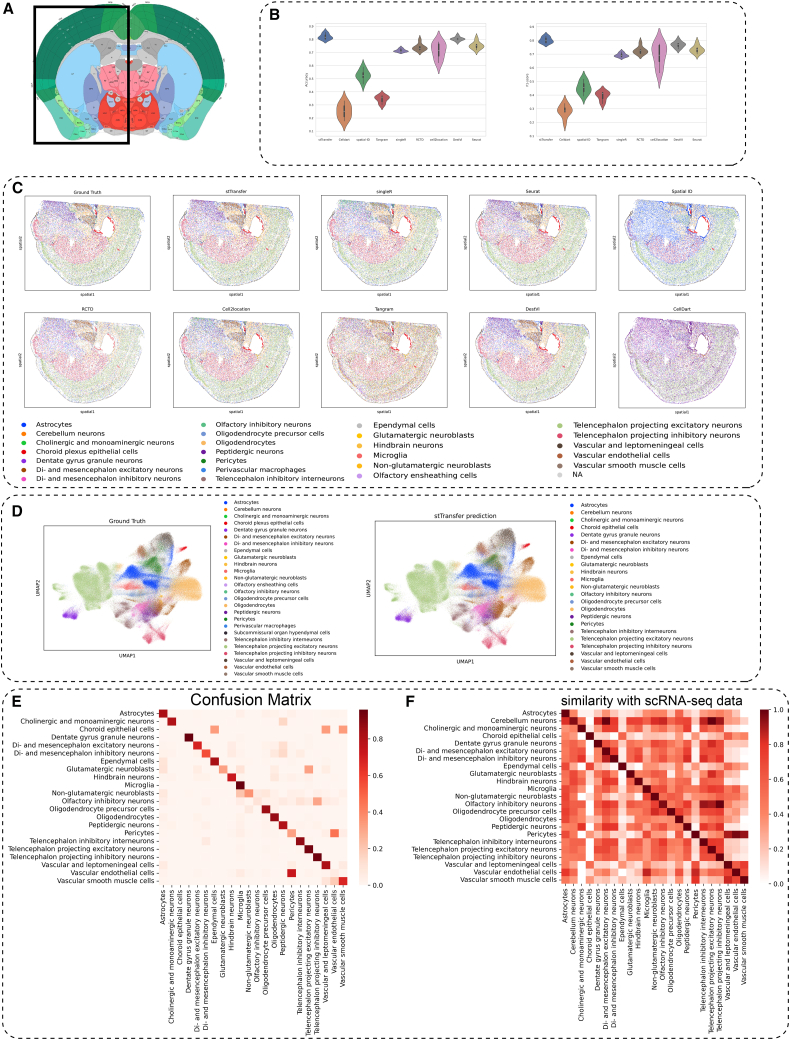
Comparison of stTransfer with other methods on mouse brain data (A) Sampling locations for the STARmap ST data. (B) Accuracy and weighted-F1 scores of different algorithm on 6 STARmap datasets. (C) Shows the spatial cell type composition of a sample ground truth, along with the cell types predicted by stTransfer and other methods. (D) UMAP visualization of ground truth cell types and stTransfer’s predicted cell types. (E) Confusion matrix illustrating the comparison between stTransfer’s predicted cell types and the ground truth cell types; the *x* axis represents predicted cell types, and the *y* axis represents true cell types. (F) Spearman correlation between stTransfer’s predicted cell types and the reference single-cell data cell types.

To rigorously assess the performance of ST annotation methods, we applied stTransfer to high-resolution STARmap data from mouse brain coronal sections,^24^ showcasing its exceptional capability in analyzing complex neural tissues. Our study focused on six randomly selected sections from the STARmap dataset, which collectively included an impressive 265,102 cells and 1,022 genes, providing comprehensive coverage of the mouse half-brain (Figure 3A). The single-cell reference dataset, sourced from the same brain region,^27^ consisted of 138,783 cells and 27,998 genes, with detailed annotations for 23 distinct neural cell types, including astrocytes, various neuronal subtypes, and vascular components.

The comparative analysis highlighted stTransfer’s outstanding performance in annotating this intricate neural dataset (Figure 3C). Across all six STARmap datasets, stTransfer achieved a mean accuracy of 82.07%, surpassing all competing methods (Wilcoxon test *p* value ≪ 0.001). stTransfer without the VAE module can achieve a mean accuracy of 63.8% on all the 6 samples. This result was further supported by its superior mean weighted-F1 score of 80.23% (Figure 3B), establishing it as the most reliable tool for annotating neural ST data.

Dimensionality reduction using uniform manifold approximation and projection (UMAP) demonstrated strong consistency between stTransfer’s predicted cell types and the ground truth cell types (Figure 3D). Additionally, the confusion matrix revealed a high level of cell type matching accuracy (Figure 3E). A robust Spearman correlation between stTransfer’s predicted cell types and the reference single-cell data cell types (Figure 3F) further validated the biological relevance and precision of the method.

In addition, we observed that the telencephalon-projecting excitatory neurons predicted by stTransfer and their corresponding marker gene SLC17A7 showed the same pattern (Figures S1A and S1B). We obtained markers for the telencephalic excitatory projection neurons predicted by stTransfer and performed Gene Ontology (GO) enrichment and found that their marker genes were mainly related to axonal information transmission (Figure S1C), aligning with the functional characterization per Shi et al.^24^

These findings underscore stTransfer’s reliability and robustness in annotating ST data within neural tissues. Its consistent performance across diverse neural cell populations, including complex groups such as telencephalon-projecting neurons and inhibitory interneurons, highlights its practical utility for studying the cellular architecture of the mammalian brain. As a result, stTransfer emerges as a powerful tool for advancing neuroscience research and ST applications.

### stTransfer delivers robust and accurate annotation of human non-small cell lung cancer ST data at single-cell resolution

To evaluate stTransfer’s performance on clinically relevant data, we applied it to a high-resolution ST dataset of human non-small cell lung cancer (NSCLC).^25^ This dataset, generated using the CosMx SMI platform with high-plex spatial molecular imaging (0.18 μm per pixel), includes 20 tissue samples comprising 83,642 cells and 980 measured genes. For reference annotation, we used single-cell data from the same tissue region,^28^ which included 49,532 cells and shared 15 common cell types with the spatial dataset. These cell types encompassed immune subsets (e.g., regulatory T, T CD8, and natural killer cells), stromal components (e.g., fibroblasts and endothelial cells), and tumor cells.

**Figure 4:**
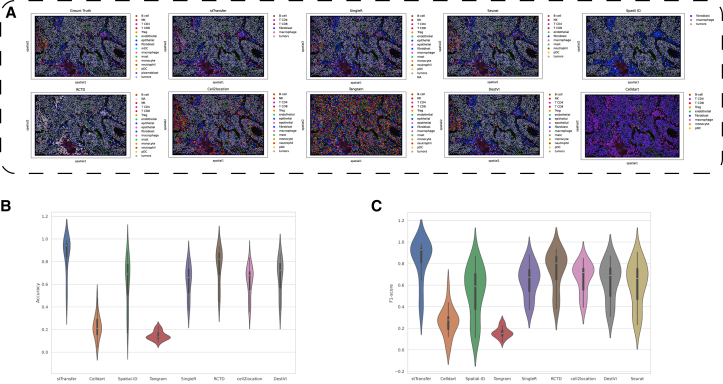
Comparison of stTransfer with other methods on human NSCLC data (A) Shows the spatial cell type composition of a sample ground truth, along with the cell types predicted by stTransfer and other methods. (B) Violin plots showing the accuracy distribution of each method across multiple samples. (C) Violin plots depicting the weighted-F1 score distribution of each method across multiple samples.

Our analysis demonstrated stTransfer’s exceptional performance on this clinical dataset (Figure 4A). The cell types predicted by stTransfer also match the histological staining. Across all 20 samples, the method achieved an impressive average annotation accuracy of 85.36% significantly surpassing competing methods (Wilcoxon *p* value ≪ 0.001) (Figure 4B). stTransfer without the VAE module can achieve a mean accuracy 62.8% on all the 20 samples. This result was further supported by stTransfer’s superior average weighted-F1 score of 82.2% (Figure 4C), underscoring its ability to consistently and accurately resolve diverse cell populations within complex tumor microenvironments.

These findings emphasize stTransfer’s robustness and precision in analyzing clinically relevant ST data, particularly in the context of human cancer tissues. Its capability to accurately annotate immune, stromal, and tumor components highlights its potential as a valuable tool for advancing cancer research and potentially guiding clinical applications.

### stTransfer demonstrates robust performance in annotating mouse spermatogenesis ST data with non-single-cell resolution

To assess stTransfer’s ability to handle ST data with non-single-cell resolution, we evaluated its performance on a mouse spermatogenesis dataset generated using Slide-seq.^26^ This dataset includes 24,105 spots and 24,105 genes, capturing nine critical cell types involved in spermatogenesis. These cell types encompass elongating/elongated spermatids, round spermatids, spermatocytes, spermatogonia, and supporting cells such as Sertoli cells, Leydig cells, endothelial cells, myoid cells, and macrophages. For reference, we used single-cell data from the same biological context, which consisted of 34,633 cells and 37,241 genes, with annotations aligned to the ST dataset.^29^

**Figure 5:**
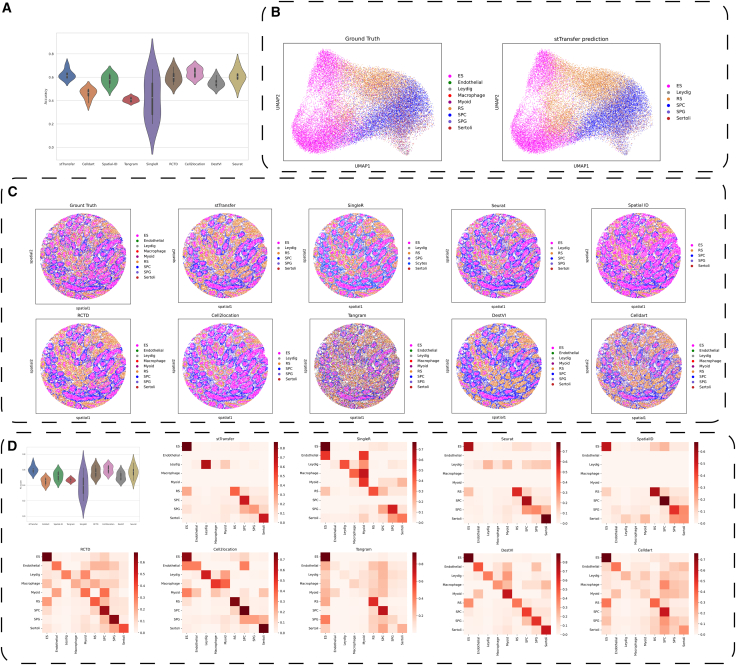
Performance of stTransfer on mouse spermatogenesis ST data (A) Violin plots showing the accuracy distribution of various methods on the mouse spermatogenesis dataset. (B) UMAP visualization of the ground truth and stTransfer’s predicted cell types. (C) Shows the spatial cell type composition of a sample ground truth, along with the cell types predicted by stTransfer and other methods. (D) Confusion matrices comparing the performance of each method against the ground truth. The *x* axis represents the predicted cell type, and the *y* axis represents the true cell type. The color scale indicates the number of cells assigned to each category.

stTransfer demonstrated strong performance on this challenging dataset, achieving the highest average accuracy of 62.43% across six samples (Figure 5A). stTransfer without the VAE module can achieve a mean accuracy of 50.6% on all the 6 samples. Although its weighted-F1 score of 60.31% was marginally lower than the 60.38% achieved by cell2location (Figure 5C), this slight difference can be attributed to the inherent complexity of the dataset. Each spot in the dataset may contain between 1 and 10 cells, a scenario that favors deconvolution-based methods. Additionally, the confusion matrix revealed that cell2location exhibited slightly better stability in resolving mixed-cell spots, underscoring the challenges associated with non-single-cell resolution data (Figure 5B).

These findings highlight stTransfer’s versatility and robustness in annotating ST data, even when single-cell resolution is not attainable. Its competitive performance in resolving complex spermatogenesis cell types underscores its potential to advance research in developmental biology and tissue-specific studies.

### stTransfer enables high-resolution spatial mapping of zebra finch OT cell types using Stereo-seq data

The OT, referred to as the superior colliculus in mammals, is a highly conserved brain structure across vertebrates and serves as a central hub for processing sensory information.^9^^,^^30^^,^^31^ It plays vital roles in visual processing, eye movement, fear responses, and prey capture. Additionally, its deeper layers integrate inputs from somatosensory and auditory systems, aligning multisensory neural maps and contributing to motor functions.^32^^,^^33^^,^^34^ Recent research has also associated the OT with higher cognitive processes, such as selective attention and decision-making.^35^^,^^36^^,^^37^ Despite millions of years of evolution, the OT exhibits remarkable similarities in its layered organization, cellular composition, and fundamental functions across species, ranging from lampreys to primates.^38^ This evolutionary conservation highlights its critical role in sensory and cognitive processing, making it an ideal model for exploring the cellular and functional architecture of vertebrate brains.

**Figure 6:**
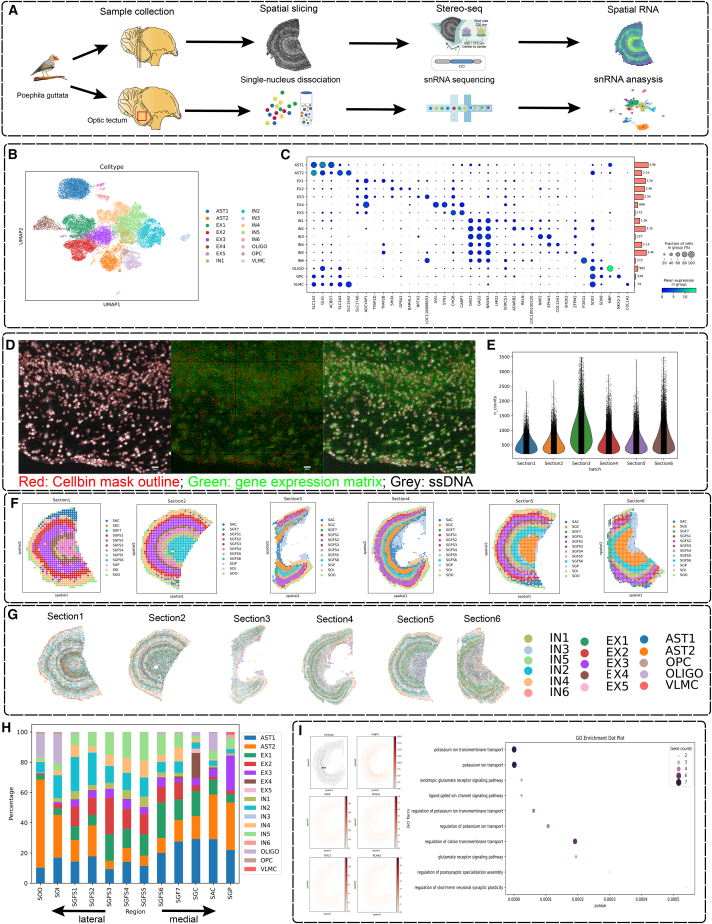
Application to zebra finch OT ST dataset sequenced by Stereo-seq (A) Zebra finch single-cell sampling pipeline and spatial transcriptome sampling pipeline. (B) Zebra finch single cell-cluster annotation UMAP plot. (C) Dotplot of marker gene and cell number distribution for each cell type. (D) Spatial transcriptome cell segmentation results; red, Cellbin mask outline; green, gene expression matrix; gray, single-stranded DNA. (E) Total counts for every section of ST data. (F) Brain region cluster annotation based on the zebra finch spatial transcriptome bin100 data. (G) Cell type annotation of every cell of ST data. (H) Cell type distribution of different region. (I) Spatial distribution of EX4 cell types in ST data and distribution of some of their marker genes, as well as GO pathway enrichment results of their marker genes.

To investigate the cellular and functional organization of the OT at high resolution, we utilized Stereo-seq, a spatially enhanced omics sequencing technology that combines high gene coverage with single-cell resolution, to study the OT of the zebra finch (*Taeniopygia guttata*) (Figure 6A). From two zebra finch brains, we generated a high-quality single-nucleus RNA sequencing (snRNA-seq) atlas of the OT, encompassing 27,489 single cells and 19,306 genes after rigorous quality control. Leiden clustering and marker gene annotation identified 16 distinct cell clusters, providing a detailed reference for spatial mapping (Figures 6B and 6C). Using Stereo-seq, we obtained ST data at single-cell resolution, capturing 103,103 spatially resolved cells across six datasets. After applying SpaGCN clustering and anatomical annotation, we integrated the snRNA-seq reference with the Stereo-seq data using stTransfer, creating a comprehensive single-cell resolution spatial atlas of the zebra finch OT (Figure 6G).

Our analysis uncovered striking spatial gradients in cell type composition across the OT. The lateral visual zone, the outermost layer, was enriched with oligodendrocytes, while the innermost periventricular gray zone predominantly contained EX3 cells expressing *NPTX2*, a gene essential for excitatory synapse formation and synaptic plasticity during development.^39^ In the central white matter layer, located between the central gray and periventricular gray layers, we observed a high concentration of EX4 cells expressing *CABP7*, *DPP6*, and *KCNG1*. GO enrichment analysis of EX4 cell markers revealed their involvement in postsynaptic specialization and short-term synaptic plasticity, potentially linking these cells to the short-term memory of visual signals in zebra finches.

This study highlights the power of combining Stereo-seq with stTransfer to resolve spatial heterogeneity at single-cell resolution, offering new insights into the cellular architecture and functional organization of the zebra finch OT. By leveraging these advanced technologies, we have uncovered critical spatial and molecular features of the OT, shedding light on its role in sensory integration and cognitive functions across vertebrates.

## Discussion

stTransfer marks a significant advancement in the field of ST, addressing the crucial gap between single-cell resolution and spatial context. This innovative method enables high-resolution annotation of cell types within their native tissue architecture by leveraging cutting-edge computational techniques. Specifically, stTransfer integrates VAEs for batch effect correction, XGBoost for transfer learning, and graph autoencoders for unified embedding, ensuring precise and context-aware cell type annotation across single-cell and ST datasets.

When applied to the zebra finch OT dataset, stTransfer unveiled biological insights. It revealed distinct spatial distributions of cell populations and their potential functional roles. For example, oligodendrocytes were found to be enriched in the lateral visual zone, while *NPTX2*-expressing EX3 cells dominated the periventricular gray zone. Additionally, EX4 cells localized in the central white matter layer showed involvement in postsynaptic specialization and short-term synaptic plasticity, potentially linking them to rapid visual memory processes in zebra finches. These findings not only deepen our understanding of the OT’s role in visual processing and synaptic plasticity but also underscore stTransfer’s ability to uncover novel biological mechanisms in complex tissues.

Despite its strengths, stTransfer is not without limitations. Key challenges include its reliance on high-quality reference datasets and the variability in spatial resolution across different technologies. These areas present opportunities for future improvements. Ongoing refinement of the algorithm, alongside advancements in ST platforms, holds promise for enhancing stTransfer’s accuracy and broadening its applicability to diverse species and tissue types.

In conclusion, stTransfer stands as a powerful and versatile tool for integrating single-cell and ST data, offering opportunities to explore the cellular and functional organization of tissues in their native spatial context. By enabling detailed mapping of cell types, stTransfer paves the way for deeper insights into tissue biology, developmental processes, and disease mechanisms, helping to advance our understanding of complex biological systems.

### Limitations of the study

stTransfer relies on a well-annotated single-cell reference and degrades when spatial resolution is low or cells are mixed; its broader utility across tissues and species remains largely untested, computational demands grow steeply with dataset size, and the graph model presumes spatial continuity that may fail in highly dispersed populations.

## Resource availability

### Lead contact

Further information and requests for the resources and reagents may be directed to and will be fulfilled by the lead contact, Shiping Liu (liushiping@genomics.cn).

### Materials availability

All materials used for Stereo-seq and snRNA-seq are commercially available.

### Data and code availability

• For the pseudo ST data application, we generated pseudo ST data and pseudo single-cell data, which can be accessed at this link (https://drive.google.com/drive/folders/1_l9N47CdlKsRgfhbHRiigmCEz1-FwEFH). For the mouse brain STARmap application, we obtained the ST data from the Broad Institute’s Single Cell Portal (SCP1830) available here (https://singlecell.broadinstitute.org/single_cell/study/SCP1830) and the single-cell data from the NCBI Sequence Read Archive under accession number SRP135960, accessible here (https://www.ncbi.nlm.nih.gov/sra/SRP135960). For the human NSCLC ST dataset application, we gathered the ST data from NanoString Technologies resource page, available here (https://nanostring.com/resources/smi-ffpe-dataset-lung9-rep1-data) and the single-cell data from the Ghent University Biomedical Research Center, accessible here (https://gbiomed.kuleuven.be/scRNAseq-NSCLC). For the mouse spermatogenesis ST dataset application, we collected the ST data from a Dropbox repository, available here (https://www.dropbox.com/s/ygzpj0d0oh67br0/Testis_Slideseq_Data.zip) and the single-cell data from the Gene Expression Omnibus under accession number GSE112393, accessible here (https://www.ncbi.nlm.nih.gov/geo/query/acc.cgi?acc=GSE112393).
• The zebra finch single-cell data used in this study can be accessed and downloaded via https://db.cngb.org/search/project/CNP0004708/. The zebra finch Stereo-seq data generated in this study can be available and downloaded via https://db.cngb.org/stomics/project/STT0000130.
• Custom code supporting the current study is available at https://github.com/zEpoch/stTransfer and https://doi.org/10.5281/zenodo.17009248.
• Any additional information required to re-analyze the data reported in this study is available from the lead contact upon request.

## Acknowledgments

The project was supported by Zhejiang Science and Technology Department (no. 2024C03004) and Hangzhou Leading Innovation Team Project (no. TD2023003).

## Author contributions

T.Z. and L.X. conceived and designed the study. T.Z. wrote the manuscript, and K.L., Y.H., Z.Z., and S.L. contributed to the discussion and revision of the manuscript. T.Z., L.X., and Y.H. provided technical support and conducted data analysis. All authors read and approved the final manuscript.

## Declaration of interests

The authors declare no competing interests.

## STAR★Methods

### Key resources table

**Table:** 

REAGENT or RESOURCE	SOURCE	IDENTIFIER
**Biological samples**		

Zebra finch	This study	N/A

**Chemicals, peptides, and recombinant proteins**		

Normal Goat Serum Blocking Solution	Vector Lab	S-1000-20
Nucleic Acid Dye	Thermo	Cat#Q10212
Qubit™ dsDNA Assay Kit	Thermo	Cat#Q10212
RNase inhibitor	NEB	M0314L
blocking buffer	Roche	Cat#11096176001
RNase A	Sigma	Cat#R4642
MgCl_2_	Ambion	AM9530G
T4 ligase	NEB	Cat#M0202V
Tissue-Tek OCT	Sakura	Cat#4583

**Deposited data**		

Pseudo spatial transcriptomics data	This study	https://drive.google.com/drive/folders/1_l9N47CdlKsRgfhbHRiigmCEz1-FwEFH
Zebra finch spatial transcriptomics data	This study	https://db.cngb.org/stomics/project/STT0000130
stTransfer	This study	https://github.com/zEpoch/stTransfer; https://doi.org/10.5281/zenodo.17009248
Zebra finch single cell data	CNGB	https://db.cngb.org/data_resources/project/CNP004708
Public mouse brain STARmap data	Broad Institute’s Single Cell Portal	https://singlecell.broadinstitute.org/single_cell/study/SCP1830
Public mouse brain single-cell data	NCBI Sequence Read Archive	https://www.ncbi.nlm.nih.gov/sra/SRP135960
Public human non-small cell lung cancer spatial transcriptomics data	NanoString Technologies	https://nanostring.com/resources/smi-ffpe-dataset-lung9-rep1-data
Public human non-small cell lung cancer single-cell data	Ghent University Biomedical Research Center	https://gbiomed.kuleuven.be/scRNAseq-NSCLC
Public mouse spermatogenesis spatial transcriptomics data	dropbox	https://www.dropbox.com/s/ygzpj0d0oh67br0/Testis_Slideseq_Data.zip
Public mouse spermatogenesis single-cell data	Gene Expression Omnibus	https://www.ncbi.nlm.nih.gov/geo/query/acc.cgi?spm=5176.28103460.0.0.297c5d27itcKqs&acc=GSE112393

**Oligonucleotides**		

Stereo-seq-TSO: CTGCTGACGTACTGAGAGGC/rG//rG//iXNA_G/	Sangon	N/A
cDNA PCR primer: CTGCTGACGTACTGAGAGGC	Sangon	N/A
Stereo-seq-library-F:/5phos/CTGCTGACGTACT GAGAGG∗C∗A	Sangon	N/A
Stereo-seq-library-R: GAGACGTTCTCGACTCA GCAGA	Sangon	N/A
Stereo-seq-library-splint-oligo: GTACGTCAGCA GGAGACGTTCTCG	Sangon	N/A
Stereo-seq-read1: CTGCTGACGTACTGAGAGG CATGGCGACCT TATCAG	Sangon	N/A
Stereo-seq-read2: GCCATGTCGTTCTGTGAGC CAAGGAGT	Sangon	N/A
Stereo-seq-MDA-primer: TCTGCTGAGTCGAG AACGTC	Sangon	N/A

**Software and algorithms**		

SAW	https://github.com/BGIResearch/SAW	6.0.1
PyTorch	https://pytorch.org/	v.1.13.1
SpaGCN	https://github.com/jianhuupenn/SpaGCN	V1.2.7
pandas	https://pypi.org/project/pandas/	V2.0.3
numpy	https://pypi.org/project/numpy/	V1.24.0
scanpy	https://scanpy.readthedocs.io/en/stable/	V1.9.3
anndata	https://anndata.readthedocs.io/en/latest/	V0.9.2
SingleR	https://github.com/dviraran/SingleR	v1.0
Seurat	https://github.com/satijalab/seurat	v5.3.0
Spatial-ID	https://github.com/TencentAILabHealthcare/spatialID	Nov 17,2022
cell2location	https://github.com/BayraktarLab/cell2location	v0.1.4
Tangram	https://github.com/broadinstitute/Tangram	v1.0.4
DestVI	https://github.com/scverse/scvi-tools	v1.3.3
CellDART	https://github.com/mexchy1000/CellDART	v0.1.1
matplotlib	https://github.com/matplotlib/matplotlib	v3.10.5
spateo	https://github.com/aristoteleo/spateo-release	v1.1.0
clusterProfiler	https://github.com/YuLab-SMU/clusterProfiler	4.3.1.900

### Experimental model and study participant details

#### Animal care

The animal protocol was approved by the Institutional Review Board School, Zhengzhou University (ZZUIRB2022-23). Animal care complied with the guidelines of this committee. One zebra finch (Taeniopygia guttata) used in this study was healthy adult at 12 months of age. Sections for Stereo-seq were from zebra finch (female).

### Method details

#### Brain tissue collection

Tissues were snap-frozen in liquid nitrogen prechilled isopentane in Tissue-Tek OCT (Sakura, 4583) and transferred to a −80°C freezer for storage before the experiment. Cryosections were cut at a thickness of 10 mm in a Leika CM1950 cryostat. Coronal segmentation was performed using a freezing microtome. Sections of zebra finch were from positions at 4.5, 4.8 and 6.5 mm from the forebrain extremity.

#### Spatial section preparation and sequencing

Stereo-seq experiment workflows were performed as previously described by Chen et al.^5^ In brief, Tissue sections were adhered to the Stereo-seq chip surface and incubated at 37°C for 3–5 min. Subsequently, the sections underwent fixation in methanol for 40 min at −20°C before initiating the Stereo-seq library preparation. Before sequencing, the same sections were stained with a nucleic acid dye (Thermo fisher, #Q10212) to detect the ssDNA distribution, and imaging was conducted using a Ti-7 Nikon Eclipse microscope before capturing them *in situ* through the FITC channel. Following this, we performed *in situ* reverse transcription, amplification, library construction, and sequencing, adhering to the manufacturer’s protocol.

#### Stereo-seq raw data processing

The fastq files from the Stereo-seq experiment underwent processing following a previously established workflow.^5^ The initial reads in the Stereo-seq data included Coordinate Identity (CID) sequences, which were aligned to the predefined coordinates of the Stereo-seq chip obtained during the first round of sequencing. During this alignment process, a maximum of one base mismatch was allowed. Reads containing Molecular Identifiers (MID) with N bases or more than two bases with a quality score below 10 were excluded from the dataset. The CID and MID associated with each read were then added to the read header. The retained reads were subsequently aligned to the reference genome using STAR,^40^ and reads with a mapping quality score greater than 10 were tallied and annotated to their respective genes. Unique Molecular Identifiers (UMIs) sharing the same CID and gene locus were merged into a single UMI, allowing for one mismatch to account for sequencing and PCR errors. This information was then used to generate an expression profile matrix containing CID information. The entire pipeline SAW can be accessed at https://github.com/BGIResearch/SAW. Notably, to improve the mapping of detected fragments to more annotated exonic and intronic regions, we generated a modified GTF annotation file based on bTaeGut1.4.pri from the National Center for Biotechnology Information (NCBI).

#### Spatial clustering and annotation

The reads captured by DNBs were summarized based on a binning method, which contained 100 3 100 single DNB in a bin100 (bin, 50 mm diameter). All bins of each section were clustered in SpaGCN (v1.2.7)^41^ separately. The average spatial position of all DNBs in one bin was set as the position of the bin, which was used in SpaGCN. Genes expressed in less than 3 bins were removed with function spg.prefilter_genes(). During spatial clustering by SpaGCN, parameters followed: *p* = 0.5, n_clusters = 28, r_seed = t_seed = n_seed = 100, and other parameters followed the official tutorial. The default clustering model of SpaGCN was used. The region annotation was based on both anatomical knowledge and unsupervised clustering results, and validated by corresponding ssDNA staining result of each section. All sections were clustered and manually annotated separately.

#### Gene ontology enrichment analysis

The top 50 DEGs with the highest average fold change and adjusted *p* value less than 0.05 of EX4 neuron were selected for the Gene Ontology (GO) enrichment analysis. Only DEGs that could be transferred into one-to-one orthologous genes of human were kept for further analysis. The GO analysis was conducted in R with a package named clusterProfiler(v4.2.2).^42^ All genes were transformed into ENTREZ ID by the human genome reference ‘org.Hs.e.g.,.db’. The compared GO enrichment was performed by function ‘compareCluster’ with paraments: ont = ‘BP’, pAdjustMethod = “BH”, pvalueCutoff = 0.05, qvalueCutoff = 0.05.

#### Image-based single-cell segmentation

To obtain the spatial data at cellular resolution, we only conducted cell segmentation on our spatial data. The cell segmentation was performed by Spateo (v1.1.0)^43^ in Python. We first identified the nucleus based on the ssDNA staining picture with a watershed-based approach. To expand the segmented cells from nuclei to cytoplasm, we then expanded each nucleus by 10 pixels and set the maximum area of each cell as 1,200 pixels.

#### Cell clustering and cell-type identification of snRNA-seq data

Basic processing and visualization of the snRNA-seq data were performed with scanpy (V1.9.3). Cells with fewer than 1,000 or more than 6,000 UMIs were excluded. Genes expressed in fewer than 5 cells were removed. After the quality control, 27,489 nuclei in total were finally remained for downstream analysis. We then integrated the snRNA-seq data from different samples with scVI^44^ with the default parameters then Principal component analysis (PCA) was performed to reduce the dimensionality to 20 components, the output embedding was applied to construct graph by *sc.pp.neighbors* with default parameters, then *sc.tl.umap* and *sc.tl.leiden* with the *resolution=0.5* were applied to data to make dimensionality reduction visualization and clustering. Degs of each celltype were calculated by the function *FindAllMarkers* in Seurat.

#### Pseudo spatial transcriptomics data generation

Two synthetic spatial datasets were generated by sampling cells from mouse brain single cell data^27^ with replacement: dataset 1 contained 4,000 non-background cells (1,000 per type, four types) and 400 background cells. Each non-background type was confined to a distinct horizontal row; background cells were distributed uniformly across the four rows; dataset 2 comprised 4,000 non-background cells (four types, 1,000 each) and 300 background cells; each non-background type occupied a distinct 100 × 100 square, whereas background cells were dispersed uniformly across the four squares.

#### Public dataset pre-process

All public datasets including single cell data and spatial transcriptomics data were pre-processed and packaged into tidy, analysis-ready AnnData objects using *anndata.AnnData* with Python.

#### Accuracy and weight-F1 score calculation

Accuracy and weighted-F1 scores were computed by comparing the cell-type labels predicted by each method against the original author-annotated labels (taken as ground truth). We used sklearn.metrics.accuracy_score for overall accuracy and sklearn.metrics.f1_score( …, average = ‘weighted') for the weighted-F1 metric, as implemented in scikit-learn.

#### Variational autoencoder for data integration

The variational autoencoder (VAE) plays a critical role in mitigating batch effects between single-cell data and spatial transcriptomics datasets. In stTransfer, the VAE module is adapted from scVI^44^ and implemented as an independent component. The input consists of gene expression matrices from both single-cell and spatial transcriptomics data, aligned by their shared gene sets. To optimize computational efficiency and enhance batch correction, we configured the latent representation dimension to 30, the hidden layer to 128 dimensions, and the decoder with two layers. This design enables the VAE to seamlessly integrate single-cell and spatial data, producing embedding matrices of consistent dimensions.

The variational autoencoder (VAE) is used to remove the batch effect between single-cell data and spatial transcriptomics data. The VAE module of this stTransfer is inherited from scVI and is independently packaged. The input data is the gene expression matrix of single-cell data and spatial group data that can be matched by the gene set. In order to obtain better computing performance and better batch removal performance, we set the dimension of latent repression to 30 dimensions, the hidden layer to 128 dimensions, and the decoder to two layers. The VAE module can integrate single-cell data and spatial data and obtain embedding matrix data with the same dimensions.

#### XGBOOST for transfer learning

Transfer learning is a machine learning technique that leverages knowledge and insights gained from one domain or task to improve performance in another related domain. In this framework, the reference dataset serves as the source domain, while the target domain comprises spatial transcriptomics data.

In Step 2 of our model, transfer learning is implemented by training an XGBoost model using the annotated embedding matrix derived from single-cell data. This process allows the spatial transcriptomics embedding matrix to learn cell type distribution patterns from the single-cell data. The XGBoost model employed here is a refined implementation of gradient boosting, specifically tailored for multi-class classification tasks. It utilizes the *multi:softmax* objective function, which assigns probabilities to each class and predicts the one with the highest likelihood. To prevent overfitting, we incorporated a dropout layer into the model architecture.

#### Establishing spatial information in spatial transcriptomics data

To capture spatial relationships among cells, we constructed a spatial adjacency graph based on the physical locations of individual cells. In this graph, nodes represent cells, and edges encode the relationships between neighboring cells. The spatial proximity between cells was quantified using Euclidean distance.

For each pairwise relationship, we computed a weight inversely proportional to the Euclidean distance using the following formula:

$$W\left(u,v\right)=e^{-\frac{d{\left(u,v\right)}^{2}}{2\theta^{2}\;}\;}$$

Here, *d(u,v)* denotes the Euclidean distance between cells u and v, and θ represents the decay coefficient. To construct the adjacency matrix, we selected the top 30 nearest neighbors for each cell. This approach ensures that spatial relationships are effectively captured while maintaining computational feasibility.

#### Autoencoder for latent representation learning

A deep autoencoder is employed to reduce the dimensionality of the cell embedding matrix *TTI*, generating a latent representation *X*. The encoder architecture consists of two fully connected layers, each followed by batch normalization, an Exponential Linear Unit (ELU) as the nonlinear activation function, and a dropout layer for regularization. The decoder mirrors this structure, featuring a single fully connected layer with identical components as those in the encoder. To ensure that the reconstructed output matrix *TTI′* closely aligns with the input matrix *TTI*, the model uses the mean squared error (MSE) loss function. This optimization process maximizes the similarity between the input and output representations.

Given input matrix $TTI\in R^{n\times d}$, the encoder generates latent representation $X\in R^{n\times k}$ through:

$$TTI_{1}=W_{1}TTI+b_{1},W_{1}\in R^{d\times k_{1}},b_{1}\in R^{k_{1}}$$

$$TTI_{1}^{BN}=BatchNorm\left(TTI_{1}\right)$$

$$TTI_{1}^{ACT}=ELU\left(TTI_{1}^{BN}\right)$$

$$TTI_{1}^{DROP}=Dropout\left(TTI_{1}^{ACT},p\right)$$

$$TTI_{2}=W_{2}TTI_{1}^{DROP}+b_{2},W_{2}\in R^{k_{1}\times k},b_{1}\in R^{k}$$

$$TTI_{2}^{BN}=BatchNorm\left(TTI_{2}\right)$$

$$TTI_{2}^{ACT}=ELU\left(TTI_{2}^{BN}\right)$$

$$X=Dropout\left(TTI_{2}^{ACT},p\right)$$

And the decoder Reconstructs $TTI^{\prime }\approx TTI$ from X:

$$X_{1}=W_{3}X+b_{3},W_{3}\in R^{k\times k_{1}},b_{3}\in R^{k_{1}}$$

$$X_{1}^{BN}=BatchNorm\left(X_{1}\right)$$

$$X_{1}^{ACT}=ELU\left(X_{1}^{BN}\right)$$

$$X_{1}^{DROP}=Dropout\left(X_{1}^{ACT},p\right)$$

$$TTI^{\prime }=W_{4}X_{1}^{DROP}+b_{4},W_{4}\in R^{k_{1}\times d},b_{4}\in R^{d}$$

The model minimizes Mean Squared Error (MSE) between input and reconstruction:

$$L_{AE}=\frac{1}{n\cdot d}\sum_{i=1}^{n}\sum_{j=1}^{d}{\left(TTI\left[i,j\right]-TTI^{\prime }\left[i,j\right]\right)}^{2}$$

#### Graph autoencoder for spatial embedding

The graph autoencoder (GAE) is designed to embed spatial neighbor graphs, which often contain a large number of nodes in spatial transcriptomics (ST) data. To enhance computational efficiency, we incorporate sparse graph convolution layers into the GAE. The graph encoder takes two inputs: the encoded representations *X* from the autoencoder and the adjacency matrix *A*. It then generates a spatial embedding S as output. The graph encoder comprises a sparse graph convolution layer, followed sequentially by a ReLU (Rectified Linear Unit) activation function, a dropout layer, and another graph convolution layer.

The final latent representations *Z* are obtained by combining the encoded representation *X* and the spatial embedding S using the formula *Z=X+S*. These latent representations are subsequently used to reconstruct both the cell embedding matrix *TTI* in the autoencoder and the adjacency matrix *A′* in the GAE. The GAE employs a cross-entropy loss function to minimize the difference between the input adjacency matrix *A* and the reconstructed adjacency matrix *A′*.

Additionally, we implement a self-supervised learning strategy to train a classifier using the final latent representations *Z* and the teacher distribution *D*. The teacher distribution *D* is derived from the cell embedding matrix *TTI*, ensuring that the model leverages high-quality reference information during training.

The graph encoder takes two inputs: the encoded representation $X\in R^{n\times k}$ (from the autoencoder) and the adjacency matrix $A\in R^{n\times n}$. It outputs a spatial embedding $S\in R^{n\times k}$:

$$S_{1}=SparseGraphConv\left(X,A\right)$$

$$S_{1}^{ReLU}=ReLU\left(S_{1}\right)$$

$$S_{1}^{DROP}=Dropout\left(S_{1}^{ReLU},p\right)$$

$$S=SparseGraphConv\left(S_{1}^{DROP},A\right)$$

The final latent representation $Z\in R^{n\times k}$ combines X and S:

Z=X+S

The GAE reconstructs $A^{\prime }\approx A$ using Z:

$$A^{\prime }=\sigma \left(ZZ^{T}\right)$$

Where σ is a sigmoid function to ensure values between 0 and 1. The GAE minimizes the difference between A and $A^{\prime }$ using cross-entropy loss:

$$L_{GAE}=-\frac{1}{n\cdot n}\sum_{i=1}^{n}\sum_{j=1}^{n}\left[A\left[i,j\right]\log\;A^{\prime }\left[i,j\right]+\left(1-A\left[i,j\right]\log\left(1-A^{\prime }\left[i,j\right]\right)\right)\right]$$

A classifier is trained using Z and the teacher distribution D. The teacher distribution D is derived from TTI:

$$D=Softmax\left(TTIW_{teacher}+b_{teacher}\right)$$

Where $W_{teacher}$ and $b_{teacher}$ are learnable parameters. The classifier minimizes the cross-entropy loss between Z and D:

$$L_{SSL}=-\frac{1}{n\cdot c}\sum_{i=1}^{n}\sum_{j=1}^{c}D\left[i,j\right]logP\left[i,j\right]$$

Where $P=Softmax\left(ZW_{student+b_{student}}\right)$ is the predicted distribution, and c is the number of classes. The total loss combines the GAE loss and the self-supervised learning loss:

$$L_{total}=W_{AE}L_{AE}+W_{GAE}L_{GAE}+W_{SSL}L_{SSL}$$

$W_{AE}$/ $W_{GAE}$/ $W_{SSL}$ are hyperparameters that could be manually setting

#### Hyperparameter settings

Totally we have 5 hyperparameters, *k_n_fold* for XGBoost training, *epochs* for AE and GAE training epochs, *w_cls* and *w_gae* and *w_dae* is the weight of different loss to caculate $L_{total}$.

#### Implementation of methods

RCTD: we used the code of RCTD from https://github.com/dmcable/spacexr, which is integrated into a tool called spacexr (2.0.0). We set doublet_mode = ‘full’. When processing the reference data, the “n_max_cells” parameter is set to 10000.

Seurat: we followed the instructions on the Seurat 3.2 Website: https://satijalab.org/seurat/archive/v3.2/integration.html. We set the parameter dim = 1:30, normalization.method = ‘SCT’, reference.reduction = “pca” for the ‘FindTransferAnchors’ function.

Tangram: we used the code of Tangram from https://github.com/broadinstitute/Tangram. We set the parameters as mode = “cells”, density_prior = ‘rna_count_based’, num_epochs = 100 for the ‘map_cells_to_space’ function.

Cell2location: we used the code of Cell2location from https://github.com/BayraktarLab/cell2location. The settings max_epochs = 250, batch_size = 2000, train_size = 1, lr = 0.002 were used for the train.

DestVI: we used the code of DestVI from https://github.com/scverse/scvi-tools. We set the parameters max_epochs = 250 when training the snRNA-seq model. The spatial model was trained for 2000 epochs, with a learning rate of 0.001.

Spatial-ID. We followed the instructions on the website: https://github.com/STOmics/SpatialID/tree/main/spatialid. When mapping labels from reference data to spatial data, we set the pca_dim = 200, k_graph = 30, edge_weight = True, epochs = 200, w_cls = 20, w_dae = 1 and w_gae = 1.

Celldart: we used the code of Celldart from https://github.com/mexchy1000/CellDART. When mapping labels from reference data to spatial data, we set num_markers = 50, nmix = 8, alpha_lr = 0.005.

SingleR: we used the code of SingleR from https://www.bioconductor.org/packages/release/bioc/html/SingleR.html for mapping labels from reference data to spatial transcriptomics data. All parameters were set to their default values during this process.

### Quantification and statistical analysis

Performance was quantified as overall accuracy and weighted-F1 score, comparing each method’s predicted cell-type labels with the author-annotated ground truth using scikit-learn’s *accuracy_score* and *f1_score( …, average=*‘*weighted*’*)*. All violin and bar plots report these metrics per sample; error bars or shaded areas denote ±s.d. across samples. Statistical significance between methods was assessed with two-sided Wilcoxon signed-rank tests across independent samples; exact *p*-values are stated in the figure legends where p ≪ 0.001 indicates significance after Bonferroni correction. Significance thresholds, error definitions, and sample numbers (n) are provided in the corresponding figure legends and Results text.

## Supplemental information

Document S1. Figure S1 and Table S1

Document S2. Article plus supplemental information
