# Supplementary material for: stTransfer enables transfer of single-cell annotations to spatial transcriptomics with single-cell resolution
Source: Cell Rep Methods. 2025 Oct 15;5(11):101205. doi: 10.1016/j.crmeth.2025.101205 (PMC12664899; doi:10.1016/j.crmeth.2025.101205)
Supplement: Document S1. Figure S1 and Table S1 [file mmc1.pdf]

Cell Reports Methods, Volume *5*

## Supplemental information

# stTransfer enables transfer of single-cell annotations to spatial transcriptomics with single-cell resolution

**Tao Zhou, Lin Xiang, Kuo Liao, Youzhe He, Zhenkun Zhuang, and Shiping Liu**

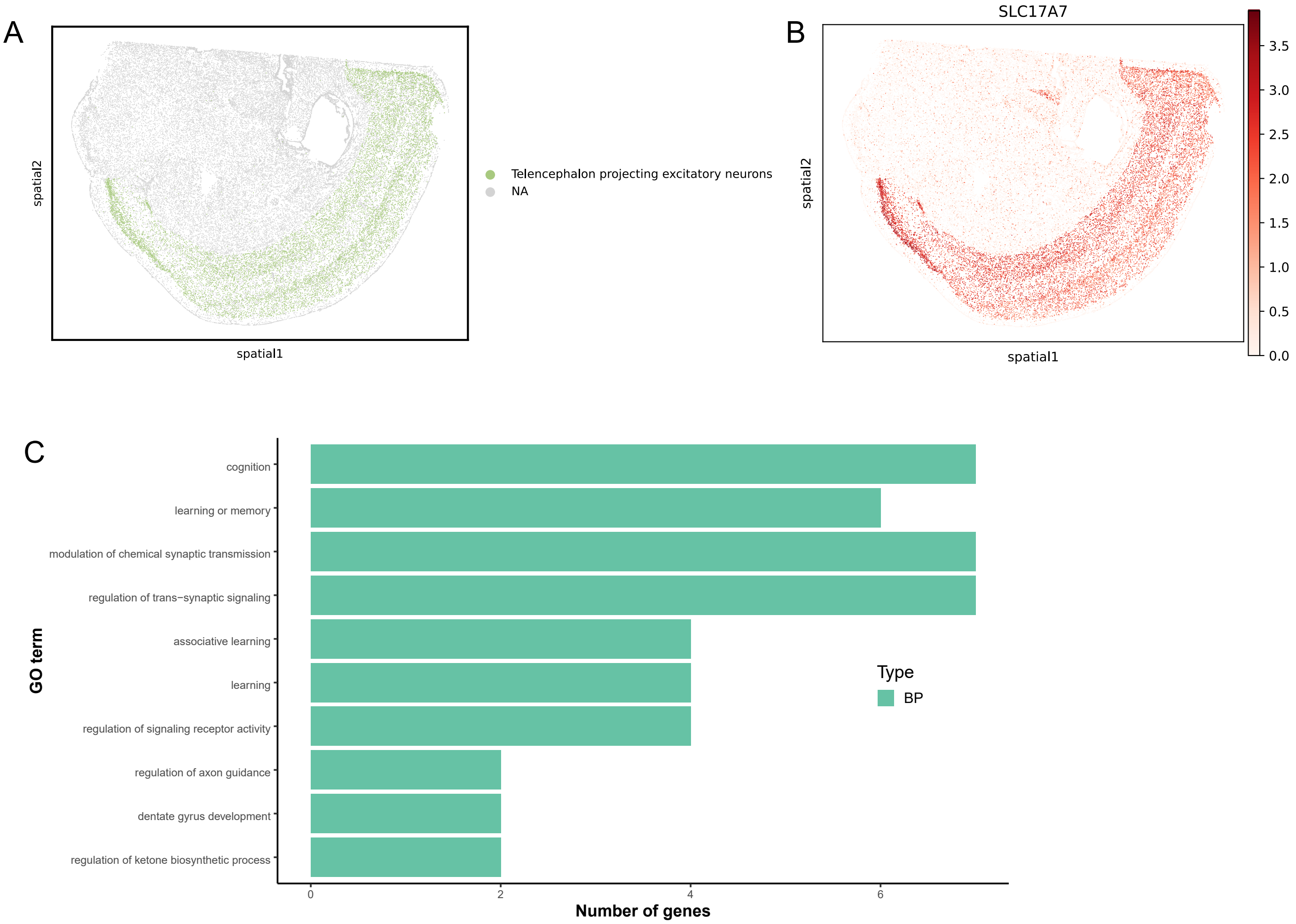

A
spatial2
spatial1
Telencephalon projecting excitatory neurons
NA
B
SLC17A7
spatial2
spatial1
3.5
3.0
2.5
2.0
1.5
1.0
0.5
0.0
C
GO term
cognition
learning or memory
modulation of chemical synaptic transmission
regulation of trans−synaptic signaling
associative learning
learning
regulation of signaling receptor activity
regulation of axon guidance
dentate gyrus development
regulation of ketone biosynthetic process
0
2
4
6
Number of genes
Type
BP

**Figure S1: Telencephalon projecting excitatory neurons and their marker genes spatial pattern display, related to figure 3.** (A) Spatial distribution of telencephalon projecting excitatory neurons in the mouse brain. (B) Spatial distribution of the marker gene SLC17A7 for telencephalon projecting excitatory neurons in the mouse brain. (C) GO enrichment of marker genes of telencephalon projecting excitatory neurons.

| SRT DATA | | | | | | Reference scRNA-seq datasets | |
|---|---|---|---|---|---|---|---|
| title | download link | sample | cells | genes | seq | title | download link |
| Chen, Haiqi, et al. "Dissecting mammalian spermatogenesis using spatial transcriptomics." Cell reports 37.5 (2021). | https://www.dropbox.com/s/ygzpj0d0oh67br0/Testis_Slideseq_Data.zip?dl=0 | 6 | 207335 | 27181 | Slide-seq | Green, C. D. et al. A comprehensive roadmap of murine spermatogenesis defined by single-cell RNA-seq. Developmental Cell 46, 651–667 (2018). | https://www.ncbi.nlm.nih.gov/geo/query/acc.cgi?acc=GSE112393 |
| He, Shanshan, et al. "High-plex imaging of RNA and proteins at subcellular resolution in fixed tissue by spatial molecular imaging." Nature Biotechnology 40.12 (2022): 1794-1806. | https://nanostring.com/resources/smi-ffpe-dataset-lung9-rep1-data/ | 20 | 91972 | 980 | CosMx SMI | Lambrechts, Diether, et al. "Phenotype molding of stromal cells in the lung tumor microenvironment." Nature medicine 24.8 (2018): 1277-1289. | https://gbiomed.kuleuven.be/scRNAseq-NSCLC |
| Shi, Hailing, et al. "Spatial atlas of the mouse central nervous system at molecular resolution." Nature 622.7983 (2023): 552-561. | https://singlecell.broadinstitute.org/single_cell/study/SCP1830 | 6 | 270320 | 1022 | STARmap | Zeisel, Amit, et al. "Molecular architecture of the mouse nervous system." Cell 174.4 (2018): 999-1014. | SRP135960 |

**Table S1: Public datasets resource, related to STAR Methods**
